# Personality Traits, Dimensions, and Suicidal Behavior in Posttraumatic Stress Disorder: Results From a Cross-Sectional Study in a Mexican Hospital

**DOI:** 10.7759/cureus.22939

**Published:** 2022-03-07

**Authors:** José C Medina, Ilyamin Merlín García, Ismael Aguilar Salas

**Affiliations:** 1 Teaching Department, National Institute of Psychiatry, Mexico City, MEX; 2 Clinical Services Department, National Institute of Psychiatry, Mexico City, MEX

**Keywords:** ptsd, personality tests, suicide, personality, posttraumatic stress disorders

## Abstract

Introduction

Posttraumatic stress disorder (PTSD) may develop after exposure to stressful or life-threatening events and is linked to suicidal behavior. Personality is hypothesized to be a mediator of this risk though assessing factors influencing such findings has been difficult due to the psychiatric comorbidities of the disorder.

Objective

The aim of the study was to examine the relationship between personality and suicidal behavior in people with PTSD.

Method

This was a cross-sectional study with 67 participants diagnosed with PTSD who completed a structured interview (SCID-II), and an inventory (NEO-FFI) to gather personality characteristics. Data were collected and analyzed through statistical software, and the investigation period ranged from August 2020 to July 2021.

Results

Extraversion was correlated with suicide attempts, borderline traits predicted the number of these, and a negative relationship was found between conscientiousness and the same variable. Schizotypal traits were also positively correlated to self-harm. Extraversion, schizoid, borderline, and obsessive-compulsive traits were more likely to be diagnosed with either depressive and/or anxious disorders. Extraversion’s association with suicidal attempts is inconsistent with previous studies, although the correlation of borderline traits with the same variable complies with existing knowledge. Schizotypal traits and their link with self-harm may be a novelty for this line of research, although the connection between extraversion, schizoid, borderline, and obsessive-compulsive traits along with depressive and anxious disorders echoes current literature.

Conclusion

Certain characteristics of personality are related to suicidal behavior in individuals with PTSD.

## Introduction

Posttraumatic stress disorder (PTSD) is a condition that may develop following exposure to stressful or life-threatening events [1]. Around 70% of adult women have been exposed to such events, and the majority of the population, regardless of gender, has undergone exposure to at least one of these during their lifetime [2]. Accompanying conditions often include mood and anxiety disorders [3]. In fact, research has shown that Major Depressive Disorder (MDD) is diagnosed simultaneously in 30% to 50% of cases while there is a lack of consensus on the comorbidity of PTSD with Recurrent Major Depressive Disorder (RMDD) [4]. Meanwhile, a report showed that anxiety disorders are some of its most prevalent comorbidities, with frequencies around 9.5% for Generalized Anxiety Disorder (GAD) followed up by 3.5% for Panic Disorder (PD) [5]. Moreover, a wide variety of neurobiological and psychosocial features associated with the disorder may influence these concurrent conditions and provide challenges for the treatment of those who are affected [6]. PTSD diagnosis is established using the criteria of the fifth edition of the Diagnostic and Statistical Manual of Mental Disorders (DSM 5), which covers intrusive, avoidant, and excitatory symptoms, as well as adverse changes of affect and cognition [7]. Its management often is difficult because of the accompanying conditions, which may include suicidal behavior [8]. This expression refers to a wide range of phenomena that encompasses characteristics such as self-harm, suicidal ideation, intent, and attempts, among other constructs [9]. In fact, one of the largest population analyses of this disorder found that individuals with PTSD had 13 times the rate of suicide than those without the diagnosis [10]. Although these antecedents offer overwhelming evidence that this condition is associated with an increased risk of suicide, some don’t take into account the underlying psychopathology [8]. Moreover, some researchers have speculated that certain comorbid conditions, like depressive symptoms and personality disorders, could be mediators of the link between PTSD and suicide [11]. Bearing in mind the latter, personality is defined as a collection of psychological traits and dimensions within individuals that are organized, enduring, and could influence their engagement with intrapsychic, physical, and social environments [12]. Traits are a set of diverse affective, cognitive and behavioral styles that might predict a multitude of psychopathological outcomes [7]. In contrast, dimensions constitute a convergence of complex psychobiological systems that interact with one another in a non-linear way [13]. Current literature holds significant knowledge about the relationship between the characteristics of personality and suicidal behavior in numerous psychiatric illnesses, though its relationship with PTSD is still inconclusive [14]. Considering this fact, the aim of this study was to examine the association between personality traits, dimensions, and suicidal behavior in individuals with PTSD.

## Materials and methods

Study design, setting, and time frame

A cross-sectional study was carried out to determine the impact of traits and dimensions of personality on the suicidal behavior of individuals with PTSD in the National Institute of Psychiatry, Mexico City, México, from August 2020 to July 2021.

Study participants

A total of 67 subjects were included using non-probability convenience sampling. Inclusion criteria encompassed male or female individuals aged 18 to 65 years being treated for PTSD under the criteria of the DSM 5. Regarding this, MDD, RMDD, GAD, and PD were permitted diagnoses due to their proven comorbidity with PTSD. Concurrently, subjects with any psychotic, bipolar, obsessive, impulse-control, neurocognitive, or substance-use disorder were excluded from the study.

Data collection

A semi-structured interview was done to gather information about sociodemographic variables. General information was collected such as age, gender, marital, occupational, and educational status. The diagnosis was assessed and based on a comprehensive clinical interview that used the diagnostic criteria of the DSM 5 intended for PTSD and the previously disclosed depressive and anxious disorders. Likewise, the screening of the excluded diagnoses was done by the method described above.

Measurements

Since personality is a multi-theoretical construct, we decided to use two instruments: one that evaluates pathological traits of personality and another that measures its dimensions. The use of both tools would provide the possibility of exploring aspects of the personality of this population from different angles and weighing their distinct outcomes. Personality traits were assessed with the Structured Clinical Interview for DSM-IV Axis II Personality Disorders (SCID-II). This is a semi-structured diagnostic interview for clinicians and researchers designed to evaluate DSM-IV Personality Disorders across Clusters A, B, and C [15]. Additionally, the Revised NEO Five-Factor Inventory (NEO-FFI), a pretested and validated instrument, was used to screen for personality dimensions. This questionnaire provides a reliable measure of the five domains of personality (Neuroticism, Extraversion, Openness, Agreeableness, and Conscientiousness) [16]. In equal measure, suicidal behavior was assessed according to the criteria of the MINI International Neuropsychiatric Interview. This validated interview features questions that assess elements such as the presence of suicidal thoughts, intent, and attempts during the last month. A slight, moderate, or serious risk of suicide was calculated with the results of this evaluation [17].

Ethical considerations

This study was conducted in accordance with the Declaration of Helsinki, no personal information was collected, and all answers were kept confidential. All participants provided written informed consent. The investigation was approved by the National Institute of Psychiatry’s Ethics Committee with certificate number CONBIOETICA-09-CEI-010-20230316.

Data analysis

Data were analyzed with the software Statistical Package for Social Sciences, version 20 (IBM Corp., Armonk, NY) [18]. Frequencies and percentages were calculated for qualitative variables like gender, marital status, occupational status, an antecedent of self-harm, previous psychiatric hospitalization, number of suicide attempts, as well as depressive and anxiety disorders. Means (M) and standard deviations (SD) were calculated for quantitative variables such as age, and years of education. A Spearman’s rank-order correlation test was computed to assess the strength of the relationship between personality dimensions and traits with variables associated with suicidal behavior (namely, suicide risk, attempts, self-harm, and an antecedent of psychiatric hospitalization). Correlation analysis was done in accordance with the normality of the data and taking into account the monotonic relationship between the study variables. Additionally, a chi-square test was conducted to examine if there was a statistical difference between the observed and expected data regarding personality traits, dimensions, depressive and anxiety disorders. Finally, a linear regression model was used to predict the influence of each personality trait and dimension on the number of suicide attempts. Relative to this, a P-value of less than 0.05 was deemed statistically significant.

## Results

All 67 participants completed the instruments that were relevant to the study, no information was lost during the planned measurements, and baseline sociodemographic and clinical data are shown in Table 1.

**Table 1:** Baseline sociodemographic and clinical data of the study population M: Mean; SD: Standard deviation; MDD: Major Depressive Disorder; RMDD: Recurrent Major Depressive Disorder; GAD: Generalized Anxiety Disorder; PD: Panic Disorder; MINI: MINI International Neuropsychiatric Interview

Variable		Gender	
		Female	Male
Total, N		59	8
Age (years), M, SD		31 ± 11	40 ± 15
Education (years), M, SD		13 ± 3	11 ± 2
Marital status, N, %	Married	15 (25.42 %)	4 (50.00 %)
	Divorced	1 (1.69 %)	0 (0 %)
	Unmarried cohabitation	3 (5.08 %)	0 (0 %)
	Widowed	0 (0 %)	0 (0 %)
	Single	37 (62.71 %)	4 (50.00%)
	Separated	3 (5.08 %)	0 (0 %)
Occupation, N, %	Employee	23 (38.98 %)	7 (87.50 %)
	Unemployed	1 (1.69 %)	0 (0 %)
	Housewife	13 (22.03 %)	0 (0 %)
	Student	12 (20.33 %)	1 (12.50 %)
	Freelancer	7 (11.86 %)	0 (0 %)
	Retired	3 (5.08 %)	0 (0 %)
MDD, N, %		17 (28.81 %)	2 (25.00 %)
RMDD, N, %		42 (71.18 %)	3 (37.50 %)
GAD, N, %		34 (57.62 %)	6 (75.00 %)
PD, N, %		9 (15.25 %)	2 (25.00 %)
Self-harm, N, %		24 (40.67 %)	3 (37.50 %)
Previous psychiatric hospitalization, N, %		14 (23.72 %)	1 (12.50 %)
Suicide attempts, N, %	One	20 (33.89 %)	0 (0 %)
	Two	4 (6.77 %)	2 (25.00 %)
	Three	1 (1.69 %)	0 (0 %)
	Four	2 (3.38 %)	0 (0 %)
Risk of suicide (MINI), N, %	None	0 (0 %)	0 (0 %)
	Slight	33 (55.93 %)	6 (75.00 %)
	Moderate	23 (38.98 %)	2 (25.00 %)
	Serious	3 (5.08 %)	0 (0 %)

The full results of the personality characteristics are shown in Table 2. Borderline traits were obtained in roughly 50% of the sample according to the SCID-II assessment. Individuals with very high neuroticism were also detected in more than half of the study population in regards to the results of the NEO-FFI inventory. When the clinical characteristics of suicidal behavior were correlated with the NEO-FFI dimensions, only suicide attempts showed a significant correlation with extraversion (r=0.306, p=0.012) and conscientiousness (r=-0.294, p=0.016). In contrast, Table 3 depicts the association of the traits of personality with the clinical variables of suicidal behavior.

**Table 2:** Personality characteristics of the study population SCID-II: Structured Clinical Interview for DSM-IV Axis II Personality Disorders; NEO-FFI: Revised NEO Five-Factor Inventory

Variable	Values		
Personality traits (SCID-II), N (%)	Paranoid		2 (3.00 %)
	Schizotypal		3 (4.50 %)
	Schizoid		5 (7.50 %)
	Histrionic		2 (3.00 %)
	Narcissistic		10 (14.90 %)
	Borderline		35 (52.20 %)
	Avoidant		2 (3.00 %)
	Dependent		3 (4.50 %)
	Obsessive-compulsive		5 (7.50 %)
Personality dimensions (NEO-FFI), N (%)	Neuroticism	Very low	0 (0 %)
		Low	2 (3.00 %)
		Moderate	11 (16.40 %)
		High	17 (25.40 %)
		Very high	37 (55.20 %)
	Extraversion	Very low	9 (13.40 %)
		Low	12 (17.90 %)
		Moderate	12 (17.90 %)
		High	22 (32.80 %)
		Very high	12 (17.90 %)
	Openness	Very low	4 (6.00 %)
		Low	16 (23.90 %)
		Moderate	19 (28.40 %)
		High	20 (29.90 %)
		Very high	7 (10.40 %)
	Agreeableness	Very low	8 (11.90 %)
		Low	13 (19.40 %)
		Moderate	19 (28.40 %)
		High	18 (26.90 %)
		Very high	9 (13.40 %)
	Conscientiousness	Very low	7 (10.40 %)
		Low	20 (29.90 %)
		Moderate	22 (32.80 %)
		High	11 (16.40 %)
		Very high	7 (10.40 %)

**Table 3:** Association of personality traits with the clinical variables of suicidal behavior R: Spearman’s rank-order coefficient; P: P-value; *: Illustrates a statistically significant Spearman rank-order correlation

Variable	Coefficient	Paranoid	Schizotypal	Schizoid	Histrionic	Narcissistic	Borderline	Avoidant	Dependent	Obsessive-Compulsive
Suicide risk	R	.021	.233	-.237	-.147	-.110	.203	-.147	.095	-.081
	P	.866	.058	.053	.237	.376	.099	.237	.446	.513
Suicide attempts	R	.005	.190	-.233	-.144	-.069	.262*	-.144	.120	-.233
	P	.967	.123	.058	.246	.581	.032	.246	.332	.058
Self-harm	R	.214	.264*	-.233	.144	.083	.055	-.144	-.031	-.118
	P	.083	.031	.057	.245	.505	.661	.245	.805	.344
Previous psychiatric hospitalization	R	.116	.230	-.153	-.094	-.124	.155	-.094	-.116	-.016
	P	.349	.061	.218	.448	.316	.210	.448	.349	.896

Concerning the research question, a positive Spearman correlation was detected between extraversion and the antecedent of suicide attempts (r=.306, p=.012). Simultaneously, a negative one was found between conscientiousness and the same variable (r=.294, p=.016). Likewise, borderline traits were also positively correlated with this factor (r=.262, p=.032). Alternatively, schizotypal traits were correlated with self-harm (r=.264, p=.031).

Table 4 displays the results of a chi-square test of independence that was conducted to study the relationship between dimensions and traits of personality together with depressive and anxiety disorders. Relating to this, participants with extraversion were significantly more likely to be diagnosed with MDD (X^2^=11.51, p=.021), RMDD (X^2^=10.56, p=.032), and PD (X^2^=12.88, p=.012). Additionally, those having schizoid traits had a higher chance to make a diagnosis of RMDD (X^2^=5.45, p=.037). Likewise, subjects with narcissistic traits had a greater likelihood to integrate GAD (X^2^=4.48, p=.033). Those with borderline traits were linked to an RMDD diagnosis (X^2^=11.43, p=.001), while obsessive-compulsive traits with both RMDD (X^2^=5.45, p=.037) and PD (X^2^=7.48, p=.028).

**Table 4:** Chi-square test for personality dimensions, traits, and their relationship with depressive and anxiety disorders MDD: Major Depressive Disorder; RMDD: Recurrent Major Depressive Disorder; GAD: Generalized Anxiety Disorder; PD: Panic Disorder; X2: Chi-square test; P: P-value; *: Illustrates a statistically significant relationship between the variables

Variable	MDD			RMDD			GAD			PD	
	X^2^	P		X^2^	P		X^2^	P		X^2^	P
Neuroticism	1.04		.793	6.02		.111	5.11	.164	1.82		.610
Extraversion	11.51		.021*	10.56		.032*	2.92	.571	12.88		.012*
Openness	4.28		.510	9.97		.076	9.37	.095	8.87		.114
Agreeableness	1.73		.785	8.63		.071	1.70	.791	3.23		.519
Conscientiousness	2.41		.661	1.74		.784	5.05	.282	4.17		.383
Paranoid	5.21		.077	.27		.552	1.39	.353	1.69		.303
Schizotypal	.04		.639	.98		.704	.06	.646	.65		.421
Schizoid	.36		.440	5.45		.037*	.87	.317	1.06		.396
Histrionic	.82		.510	1.01		.448	.08	.647	.40		.697
Narcissistic	.02		.585	1.57		.186	4.48	.033*	.11		.520
Borderline	2.52		.094	11.43		.001*	2.08	.116	3.29		.068
Avoidant	.47		.490	.27		.552	3.05	.159	1.69		.303
Dependent	.04		.639	.98		.704	.06	.646	.62		.579
Obsessive-compulsive	.36		.440	5.45		.037*	.92	.324	7.48		.028*

Finally, the only notable finding in our regression analysis was that borderline traits were predictors of the number of suicide attempts was. Hence, no other predictors were detected with the remaining study variables while using this model. Considering this, a significant regression equation was found (F (1,65) =4.696, P<.034) with an R^2^ of .053. Thus, the participant’s predicted number of attempts was equal to .375 + .482 (borderline traits) * suicide attempts in cases where these traits were present. In simple terms, the person’s suicide attempts increased by .482 when these factors were diagnosed.

## Discussion

The current study aimed to assess the relationship between traits and dimensions of personality with suicidal behavior in individuals with PTSD. First and foremost, the majority of the sample were women, and the only significant factors positively associated with the antecedent of suicide attempts were extraversion and borderline traits while conscientiousness was found to be negatively linked. Regarding extraversion, this finding is interesting considering that a recent multi‐cohort study found no clear evidence of it being related to suicide rates in any of its analyses [19]. By contrast, borderline traits have been heavily associated with suicidal attempts, which our study also replicated in regression analysis [20]. Whereas, deficits of conscientiousness have proven to predict suicide likelihood in other studies [21-22]. This is not surprising, given that this factor suggests the tendency to be self-controlled, organized, and rule-abiding [23]. On the other hand, our correlation analysis also demonstrated a significant relationship between schizotypal traits and self-harm. Such a finding is also interesting, bearing in mind that a recent report found that self-harm was associated with psychotic traits, without specifically addressing schizotypal ones [24]. In fact, past research has shown that people with these traits are at increased risk for social withdrawal and isolation, which might explain this association [25]. Furthermore, comorbid psychiatric illness was also closely related to these features of personality. Extraversion was found more frequently in subjects with MDD, RMDD, and PD. This is inconsistent with previous literature and should be explored in further studies, considering that this dimension negatively predicts this type of psychopathology, possibly through its capacity to prevent social dysfunction [26]. Likewise, schizoid traits had a higher chance to be diagnosed in parallel with MDD while narcissistic ones with GAD. Pertaining to this, cluster A traits have consistently proven to be related to general psychopathology (including depression) and possibly due to low social interaction [27]. Conversely, narcissistic traits are demonstrated predictors of both state and trait anxiety, a finding that ends up being in line with the results of this work [28]. Moreover, borderline, and obsessive-compulsive traits were closely tied to an RMDD diagnosis while the latter was also related to PD. This also echoes current literature since the former traits have always been linked to depressive disorders while the latter with both anxiety and intolerance to uncertainty [29-30]. Finally, no significant differences for the other variables’ scores or correlations were found. Though, it must be noted that no participant had zero suicide risk and that this finding might be due to the coexisting characteristics described earlier. In summary, our results show that some features of personality are associated with a myriad of elements relating to the suicidal behavior of individuals with PTSD. This finding helps corroborate existing literature’s evidence that personality will not only influence the development of such behavior but may also positively predict depressive or anxious psychopathology. It could be argued that this disorder could act as a modifier of the typical or expected course of certain personality characteristics, and this may elucidate some of the divergences found in this study. Though, in a wider context, it needs to be considered that most past studies have focused solely on the interaction between personality and suicidal behavior, without regard to comorbid PTSD. Given this, longitudinal research may be warranted to better understand the long-term implications of this factor in the suicidal behavior of this patient population.

Limitations

Notwithstanding several important results of this research, some limitations must be considered. First, our research was done using a cross-sectional design, which is not able to provide compelling evidence for causality. That said, it is difficult to infer the point at which the personality characteristics of this population were generated. Further, the study framework did not consider the type of traumatic event experienced by the participants nor the moment in time in which it occurred. Perhaps a model that compares the type of trauma, traits, dimensions, as well as their formation over time, could elucidate more about the precise differences surrounding their link with PTSD. Second, the use of a self-administered questionnaire such as the NEO-FFI could favor a recall bias. Third, the Hawthorne effect cannot be ruled out. That is, the participants may have acted differently since they knew that they were in experimental work. Fourth, selection bias cannot be dismissed, given that only participants who receive treatment at the National Institute of Psychiatry were able to take part in the study. Even though the sample size was sufficient, the type of selection technique that was utilized meant the size could not be generalized for the general population. Given this, these assumptions can carry a risk of having an impact on the external validity of the investigation. Nonetheless, the current report adds value to the current literature since few studies have focused on both dimensions and traits of personality amongst people diagnosed with PTSD. This might even be more significant since little research emphasizes its associated impact on suicidal behavior. Considering this, future studies will be necessary to better understand this phenomenon.

## Conclusions

Altogether, our results suggest that specific characteristics of personality are associated with various outcomes that involve the suicidal behavior of individuals diagnosed with PTSD. Extraversion and borderline traits were correlated with suicide attempts, the latter were significant predictors of the number of attempts and schizotypal ones were associated with self-harm. Likewise, extraversion, schizoid, borderline, and obsessive-compulsive traits were correlated with depressive and anxiety disorders. Bearing in mind these points, the findings of this research have shed some light on the effects of the traits and dimensions of personality in certain components of suicidal behavior and accompanying psychiatric illness in individuals diagnosed with this disorder. However, analytical research will be required to better understand this phenomenon. Given this, we call for the implementation of adequate measures to raise awareness about the study of personality and suicide in individuals diagnosed with this condition.
